# High temperature gas sensing performances of silicon carbide nanosheets with an n–p conductivity transition†

**DOI:** 10.1039/c8ra02164c

**Published:** 2018-04-12

**Authors:** Lian Sun, Cheng Han, Nan Wu, Bing Wang, Yingde Wang

**Affiliations:** Science and Technology on Advanced Ceramic Fibers and Composites Laboratory, National University of Defense Technology Changsha 410073 China cfcwb01@163.com wangyingde@nudt.edu.cn +86 731 84574196

## Abstract

Fast and effective detecting of flammable and explosive gases in harsh environments (high temperature, corrosion atmosphere) is crucial for preventing severe accidents for the chemical industry, fuel cell applications and engine tests. Silicon carbide material is reported to be a good candidate for gas sensing devices applied in extreme conditions. Herein, high-temperature available silicon carbide nanosheets (SiC NSs) were synthesized from graphene oxide (GO) *via* a catalyst-free carbothermal method. The structure and composition of SiC NSs under different reaction conditions are carefully characterized. The received SiC NSs were firstly utilized as gas sensing materials for hazardous gases (acetone, ethanol, methanol and ammonia) at a high temperature (500 °C). Importantly, the SiC NSs sensors exhibited a fast response (8–39 s) and recovery (12–69 s) towards detecting gases. Besides, an n–p conductivity transition phenomenon is found and studied. This paper firstly proves that such SiC NSs has the potential to be used in gas sensing fields.

## Abstract 2

Novel silicon carbide nanosheets were synthesized by a carbothermal reduction reaction. We studied their high-temperature gas sensing properties and the mechanism of n–p conductivity transition during gas sensing tests.

## Graphical abstract

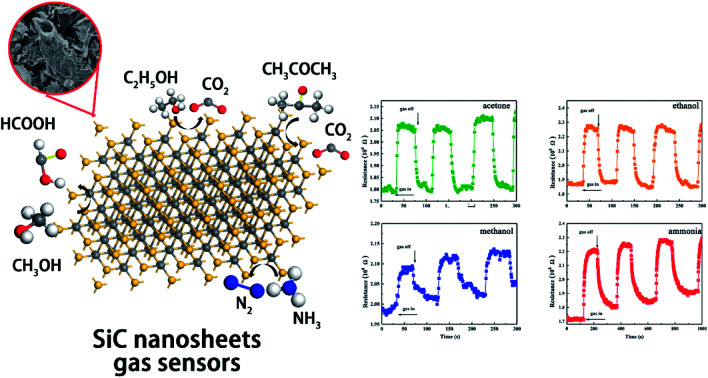

## 1. Introduction

Recently, many industrial fields, such as controlling combustion processes,^1^ chemical synthesis,^2^ turbine engine tests^3^ and the car industry,^4^ require *in situ* environmental monitoring devices that can be applied in harsh environments (high temperature, corrosive atmosphere, *etc.*). Particularly, the leakage of flammable and explosive gases (ethanol, ammonia and methanol) may cause huge accidents at high temperature if people cannot find them in time.^5–7^ For example, the typical operating temperature of ethanol fueled solid oxide fuel cells (SOFCs) is 500–1000 °C.^8^ Thus, it is necessary to develop novel gas sensors that can be used in extreme conditions with rapid response/recovery time. In the past decades, gas sensors based on metal oxides,^9,10^ graphene^11,12^ and polymers^13,14^ have been widely investigated due to their low cost, high carrier mobility and excellent designability. However, the long response/recovery time and not satisfactory performance at high temperature severely restrict their wide applications. Therefore, novel materials which can keep good properties under harsh environments are urgently needed.

As a third-generation wide band gap semiconductor, silicon carbide (SiC) has emerged abundant interests because of its excellent chemical inertness and good capability with existing silicon-based electro devices.^15–17^ Besides, SiC has been proven as a qualified candidate for the application in extreme condition,^18,19^ and its high charge transport rate makes it possible to fabricate gas sensors with short response/recovery time.^20^ Soo *et al.* demonstrated that SiC material is the best candidate for some particular situations (chemical synthesis, vapor-turbine tests, *etc.*) requiring gas sensing materials that can be used under 500 °C.^2^ Hence, using SiC to design high-performance gas sensors applied into harsh environments is extremely expected. In the past decades, scientists have investigated large amount of SiC-based metal-oxide-semiconductor (MOS) gas sensors.^21–23^ Recently, nanostructure SiC gas sensors have also been studied widely.^24–27^ Compared with other nanostructures, two-dimensional (2D) nanostructure may perform better on gas sensing due to its special characteristics, such as high surface area, large active sites and high charge carrier mobility,^28–32^ which may benefit the absorption of gas molecules and promote the transport of electrons. Besides, it has been proven that the electronic structure of 2D SiC can be adjusted easily, making it possible to be used in various electronic devices including sensors.^33,34^ Thus, combining the advantages of 2D materials with SiC is expected to fabricate high-performance gas sensors which can be applied in harsh environments. Dong *et al.* reported that the novel SiC_5_ siligraphene could form stable absorption with air pollution molecules, including NO, HCHO and SO_2_*via* density functional investigations (DFI).^35^ But to the author's knowledge, no relevant papers have synthesized actual products to test the gas sensing properties of two-dimensional silicon carbide nanostructures.

In this work, we successfully prepare novel silicon carbide nanosheets (SiC NSs) *via* a simple carbothermal reduction reaction. The effects of heating time and temperature to the structure and composition of SiC NSs have been carefully studied using different characterization methods. Such SiC NSs possess good response towards hazardous gases (acetone, methanol, ethanol and ammonia) under 400–500 °C, which is possible to make them applied into harsh environments. The SiC NSs gas sensor also shows faster response/recovery time towards ethanol than most reported ethanol-sensing materials. Besides, an n–p conductivity transition phenomenon is found and detailedly investigated. This study opens a new direction for controlling the synthesis of SiC NSs *via* carbothermal reduction method and applying SiC-based materials into gas sensing field.

## 2. Experimental section

### 2.1 Reagents

Silicon powder (Si), hydrofluoric acid (HF), hydrogen peroxide (H_2_O_2_, 30%), potassium permanganate (KMnO_4_), phosphoric acid (H_3_PO_4_), nitric acid (HNO_3_) and sodium bicarbonate (NaHCO_3_) were provided by Sinopharm Chemical Reagent Co., Ltd (Shanghai, China). Sulfuric acid (H_2_SO_4_) and hydrochloric acid (HCl) were acquired from Zhuzhou Xingkong Glass Co., Ltd (Zhuzhou, Hunan, China.). Natural flake graphite powder and ethanol were purchased from Tianjin Kermel Chemical Reagent Co., Ltd (Tianjin, China). All the chemical reagents were used as received.

### 2.2 Preparation of graphene oxide (GO)

GO was prepared following a modified Hummers' method.^36^ Typically, 3.0 g graphite powder was dissolved in a mixture acid containing concentrated H_2_SO_4_ (360 ml) and H_3_PO_4_ (40 ml) under stirring in an ice bath. Then 18 g KMnO_4_ was slowly added to the solution. After that, the whole system was transferred to a 35 °C water bath and kept stirring for 2 h. After that, the temperature was increased and kept 60 °C for another 12 h. Then 20 ml H_2_O_2_ was slowly added until the brown solution turned to yellow. The mixture was centrifugated and washed by 10 vol% HCl solution and deionized water successively. After that, ethanol was used to wash repetitively until no GO was dissolved into ethanol. The resulting GO/ethanol solution was freeze dried using the system provided by Beijing Songyuan Huaxing Technology Develop Co., Ltd (Beijing, China) to obtain the final product.

### 2.3 Preparation of SiC NSs

**Scheme 1:**
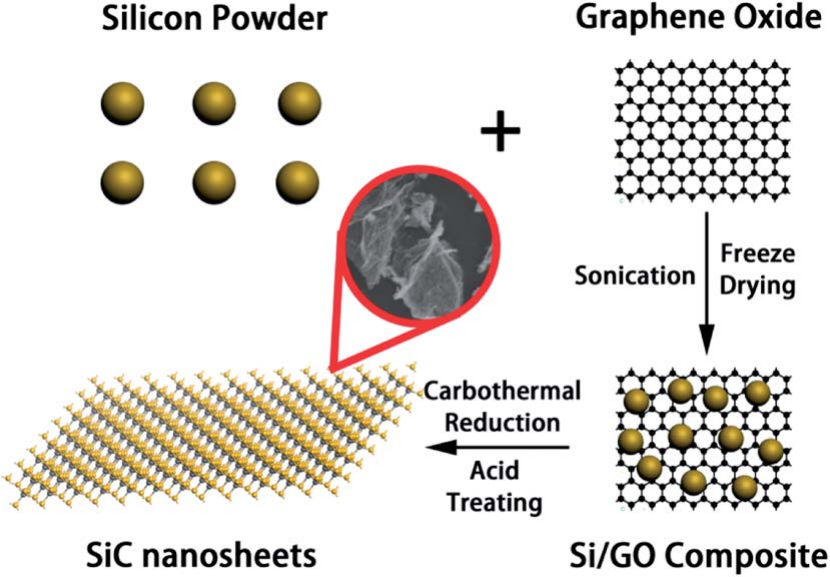
Synthetization process of SiC NSs.

The whole preparation process of SiC NSs is illustrated in Scheme 1. To prepare SiC NSs, 0.1 g GO was firstly dissolved to 30 ml deionized water and sonicated for 2 h until all the solid was dispersed. Then 0.3 g Si powder was added to the solution (the mass ratio of Si : GO was 3 : 1) and the mixture was continued to sonicate for additional 0.5 h. The sample was freeze dried to form Si/GO composite. After that, the composite was put into the center of a tube furnace and heated at 1300–1500 °C for 0.5–3.0 h with an argon flow. Typically, the reaction conditions are summarized in Table 1. At the end of the preparation process, the resulting sample was dipped into a mixed acid consisting of HF (10 ml) and HNO_3_ (30 ml) for 6 h to remove the excess silicon, washed for several times using deionized water and dried under 80 °C in a vacuum drying oven overnight. In order to distinguish different samples more clearly, all the SiC NSs are denoted as SiC-*x*_*y*_, where *x* and *y* is the carbothermal reaction temperature and heating time, respectively.

**Table 1:** Different reaction conditions of SiC NSs

Sample	SiC-1300_2.0_	SiC-1400_0.5_	SiC-1400_1.0_	SiC-1400_2.0_	SiC-1400_3.0_	SiC-1500_2.0_
Temperature (°C)	1300	1400	1400	1400	1400	1500
Duration (h)	2.0	0.5	1.0	2.0	3.0	2.0

### 2.4 Characterization

To observe the morphology and structures of our samples, we used Hitachi S-4800 field-emission scanning electron microscope (FE-SEM, Chiyoda, Tokyo, Japan) with an acceleration voltage of 10.0 kV and JEM-2100HR transmission electron microscope operating at 200 kV (TEM, JEOL Ltd., Akishima, Tokyo, Japan). X-Ray diffraction (XRD) data were recorded on Bruker AXS D8 Advance device (Billerica, MA, Cu-Kα *λ* = 1.5418 Å) working at 40 kV and 40 mA (2*θ* = 0.02° per step). X-Ray photoelectron spectroscopy (XPS, Thermo Scientific ESCALAB 250Xi spectrometer Al-Kα, Waltham, Massachusetts, USA) was obtained to analyze the composition and existing status of elements. In order to investigate the structures more detailedly, Fourier Transform Infrared spectra (FTIR, Nicolet Avatar 360, Nicolet, Waltham, Massachusetts, USA, KBr pellets) and Raman spectroscopy (LABRAM HR, Horiba, Kyoto, Japan) data were also collected. Thermo Gravimetric Analysis (TGA) was performed on Pysis 1 (PerkinElmer, Waltham, MA, USA) system in air with a heating rate of ∼10 °C min^−1^. The Mott–Schottky curve measurements were performed in a typical three-electrode electrochemical system (CHI 760e, Chenhua, Shanghai, China) at room temperature using Pt wire as the counter electrode and SCE as the reference electrode.

### 2.5 Gas sensing test

The gas sensing test was performed on a common-used intelligent gas sensing analysis system (CGS-1TP, Beijing Elite Tech Co., Ltd., Beijing, China).^37,38^ The gas sensing devices were fabricated using commercial aluminum oxide substrates with Au interdigitated electrodes (10.00 mm × 5.00 mm × 0.25 mm, Aurora Technologies Co,. Ltd. Guangzhou, Guangdong, China). A schematic illustration of the gas sensing device and testing system was shown in Fig. S1 in ESI.† First, the prepared sample was dissolved into deionized water (10 mg ml^−1^) to obtain a homogeneous paste. Then the paste was coated on the electrode and the device was subsequently dried naturally in air for several hours. Before gas sensing test, the electrode was put under the probes to form electrical contact. The operating temperature can be controlled from room temperature to the target. After the resistance of electrode remained stable, corresponding amounts of target gas was injected into evaporator (for liquid) or directly in the test chamber (for gas, 18 L in volume). During the whole testing process, the temperature of evaporator was set as 120 °C. Finally, the test chamber was opened to make the impedance of sensor recover.

Here, the sensor response (*S*) is defined as:1
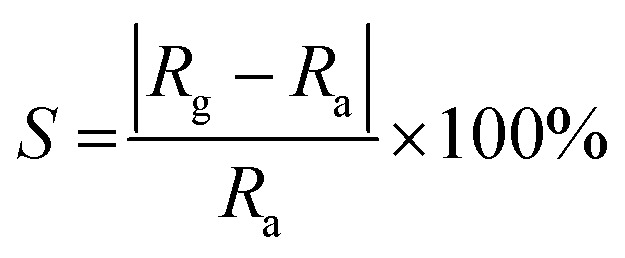
where *R*_g_ and *R*_a_ are the resistances of sensor in target gas and air, respectively. The response/recovery time is calculated by using the time taken by the sensor to experience 10–90% of the steady state resistance when target gas/air is introduced.

## 3. Results and discussion

### 3.1 Characterization

**Fig. 1:**
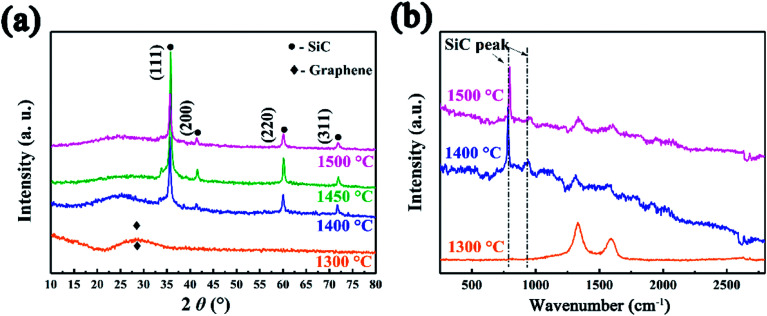
(a) XRD patterns and (b) Raman spectra SiC NSs synthesized under different temperatures for 2 h, showing the forming of SiC only after 1400 °C.

The SiC NSs were produced by direct carbothermal reduction reaction between graphene oxide and silicon powder. Fig. 1 shows the XRD patterns and Raman spectra of the resulting SiC NSs heated under different temperatures for 2 h (SiC-1300_2.0_, SiC-1400_2.0_ and SiC-1500_2.0_, respectively). It can be clearly seen that the silicon carbide phase in samples only forms above 1400 °C. The typical diffraction peaks of samples heated at 1400 and 1500 °C at 2*θ* = 35.6, 41.2, 60.2 and 72.1° correspond to the (111), (200), (220) and (311) lattice of β-SiC, respectively [JCPDS card (29-1129)], revealing that the composition of resulting SiC NSs is mainly β-SiC.^39,40^ The weak peak appearing around 2*θ* = 33.6° (S. F. in Fig. 1a) may be originated from the stacking faults along (111) plane.^41^ The wide peak at around 2*θ* = 26.1° is caused by the amorphous SiO_2_ existing in the SiC NSs. On the contrary, SiC-1300_2.0_ only gives a wide peak at around 23.2°, which belongs to the diffraction peak of reduced graphene oxide (rGO).^42^ The SiC peaks (796 and 954 cm^−1^) in Raman spectra (Fig. 1b) also appear only when the heating temperature is above 1400 °C, matching well with XRD patterns. It is worth noticing that both D and G peaks (1337 and 1599 cm^−1^, respectively) disappear above 1400 °C. Since those typical peaks always reflect the status of carbon atoms in graphene, this result may indicate that the original structure of GO has been broken after carbothermal reduction reaction. Similarly, the FT-IR spectra (Fig. S2†) only give peak of Si–C band (760 cm^−1^) after 1400 °C. The disappearance of peaks belonging to hydroxyl group (3400 cm^−1^) and C–C double bond (1620 cm^−1^) may be caused by the trace amount of residual rGO phase.

**Fig. 2:**
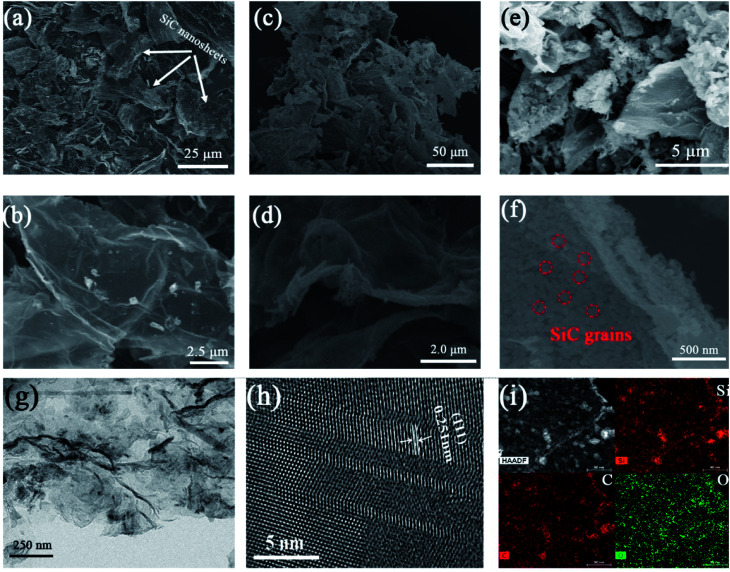
SEM images of SiC NSs synthesis under (a) and (b) 1300 °C, (c) and (d) 1400 °C and (e) and (f) 1500 °C. (g) and (h) TEM images and (i) element mapping distribution of SiC NSs heated under 1500 °C.

SEM and TEM were employed to clearly observe the morphology and structures of different samples. Fig. 2 shows the SEM images of SiC-1300_2.0_, SiC-1400_2.0_ and SiC-1500_2.0_. The sonication process produces large number of fragments (Fig. 2a, c and e), and the shape of nanosheets does not change obviously after carbothermal reduction (Fig. 2b, d and f). It should be noted that at 1500 °C, a plenty of SiC grains can be observed on the GO template (Fig. 2f). This is mainly because that the growth rate of crystal particle is fast under high temperature. TEM image of SiC NSs heated at 1500 °C (Fig. 2g and h) indicates that the interplanar spacing of the lattice fringes is 0.251 nm, which belongs to the (111) lattice of 3C–SiC.^43^ The element mapping results of SiC NSs heated at 1500 °C is also given in Fig. 2i. The Si and C elements are both homogeneously distributed. Meanwhile, the O element can be also seen on the surface, which may suggest that an oxide layer may be formed on SiC NSs.

**Fig. 3:**
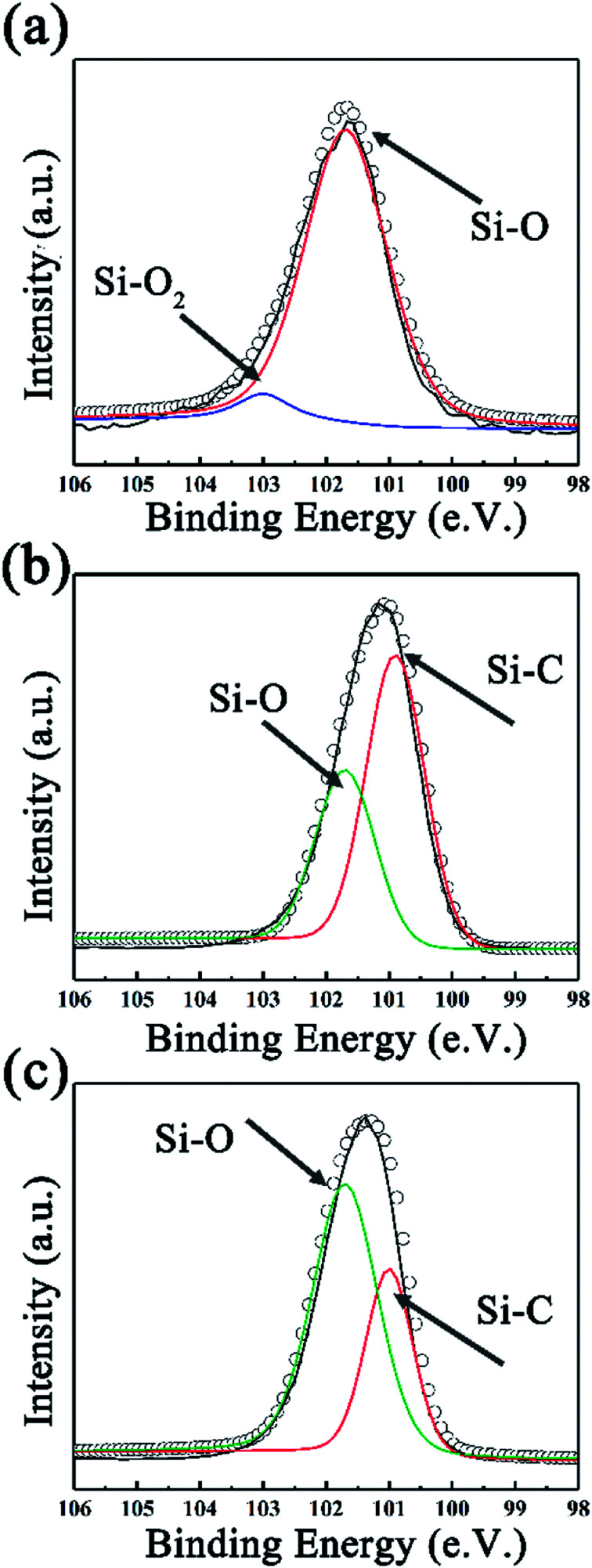
The fitted Si_2p_ spectra of SiC NSs synthesized at (a) 1300, (b) 1400 and (c) 1500 °C.

To further investigate the surface environment change, XPS data were analyzed by XPSPEAK 4.1 in order to reveal the variation of element status. As can be seen in Fig. 3a, the deconvolution of Si_2p_ peak of SiC-1300_2.0_ gives peaks at 101.7 and 103.1 eV, which can be ascribed to Si–O and Si–O_2_ bond, respectively.^44^ Considering that no silicon-relevant phase can be found in XRD, Raman and FT-IR results, this may indicate that trace of silicon powder is absorbed on the surface of GO *via* oxygen-containing functional groups on that. Besides Si–O, an Si–C peak can be found in the high resolution Si_2p_ peak of SiC-1400_2.0_ and SiC-1500_2.0_ (ref. 45 and 46) (Fig. 3b and c). The detailed information of the deconvolution of Si_2p_ peak is provided in Table S1 in our ESI.† The deconvolution of C_1s_ and O_1s_ peaks are also provided in Fig. S3 (ESI†), which could further indicate the formation of Si–C over 1400 °C.

**Fig. 4:**
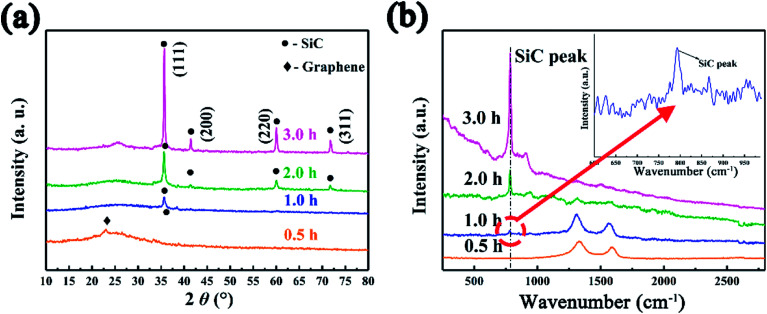
(a) XRD patterns and (b) Raman spectra of SiC NSs heated under 1400 °C for different time. Inset of (b) is the zoom Raman spectrum of SiC NSs heated for 1.0 h, suggesting the successful formation of SiC.

**Fig. 5:**
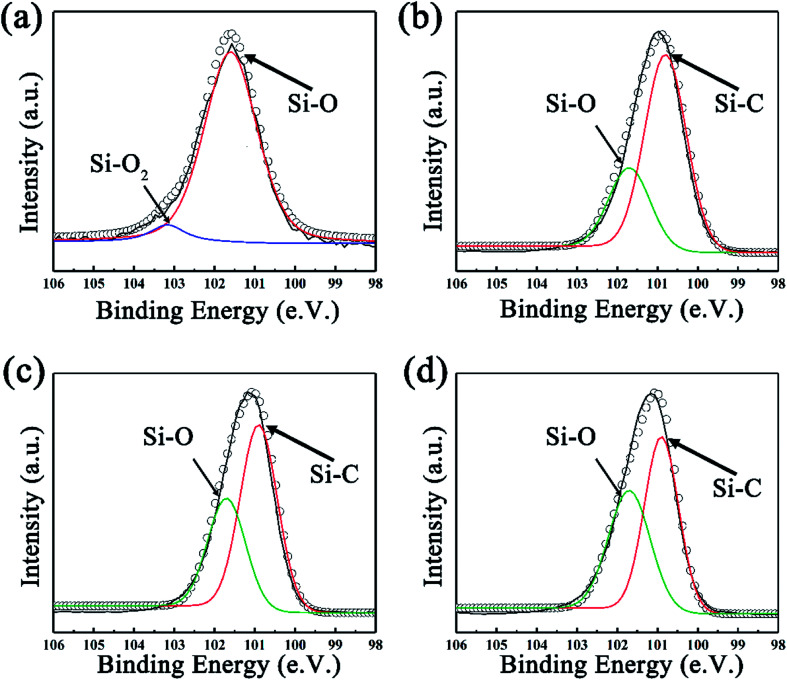
The fitted Si_2p_ XPS spectra of SiC NSs synthesized at 1400 °C for (a) 0.5 h, (b) 1.0 h, (c) 2.0 h and (d) 3.0 h.

The effect of heating time to the morphology and structure of SiC NSs has also been detailedly studied. The reaction time has been carefully controlled to 0.5–3.0 h under 1400 °C (SiC-1400_0.5_, SiC-1400_1.0_, SiC-1400_2.0_ and SiC-1400_3.0_). The XRD pattern (Fig. 4a) shows that no SiC composition can be formed when the heating time is 0.5 h. The diffraction peaks of SiC become sharper gradually with the increase of reaction time, indicating the high crystallinity under long heating. Raman (Fig. 4b) and FT-IR (Fig. S4†) spectra also prove the forming of SiC phase after heating 1.0 h. However, it is worth noticing that the D and G peak still exist after heating 1 h, which may be because of the influence of residual carbon phase. The surface morphologies of SiC-1400_0.5_, SiC-1400_1.0_, SiC-1400_2.0_ and SiC-1400_3.0_ were clearly observed by SEM. As can be seen in Fig. S5,† all the samples keep a good sheet-like structure regardless of the carbothermal reaction. Particularly, it is clearly found that many SiC NSs fragments stack each other, obtaining multi-layer structure, which may generate tunneling effect and improve their electrical properties. XPS analysis was also utilized to further characterize the change of chemical composition. Fig. 5 illustrates the high resolution Si_2p_ peak of SiC-1400_0.5_, SiC-1400_1.0_, SiC-1400_2.0_ and SiC-1400_3.0_. Similar with the above results, only Si–O–C and Si–O_2_ bond are shown in the high resolution Si_2p_ peak of SiC-1400_0.5_, while a Si–C peak is given in that of other samples (for details, see Table S1 in ESI†). The deconvoluted C_1s_ and O_1s_ peaks are also given, as shown in Fig. S6.† No Si-bonds can be found in the deconvolution of C_1s_ and O_1s_ peaks of samples heated for 0.5 h, corresponding to the XRD, Raman and FT-IR results.

The element contents were estimated using integrated areas and relative sensitive factor (RSF) of each element:^47^2
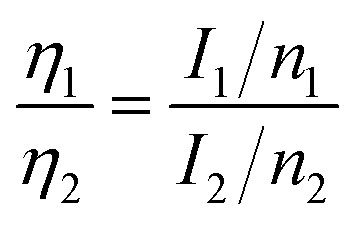
where *η*_i_ was the relative amount of the atom, *I*_i_ was the integrated peak area and *n*_i_ was the RSF. To summarize the effect of heating temperature and time to the composition of SiC NSs, the element content of all above six samples were shown in Table 2. To sum up, the SiC phase only forms above 1400 °C, and the minimum heating time is 1.0 h. As the heating time increases, the carbon amount will decrease, resulted from the transfer to SiC of grapheme oxide. However, the stoichiometric ratio of C to Si is not always 1 : 1 because of the residual rGO inside SiC NSs, which can be reflected by the Thermo Gravimetric Analysis (TGA) results (Fig. S7†). To directly show the variation of elements in different samples, the XPS survey peak of samples are summarized in Fig. S8,† matching well with the results in Table 2.

**Table 2:** The element content of all SiC NSs samples (atomic%)

Sample	SiC-1300_2.0_	SiC-1400_0.5_	SiC-1400_1.0_	SiC-1400_2.0_	SiC-1400_3.0_	SiC-1500_2.0_
Si	7.38	7.02	21.8	40.67	42.85	40.09
C	88.43	89.67	67.11	47.84	46.31	47.36
O	4.19	3.31	11.09	11.49	10.85	12.55

### 3.2 Gas sensing properties and possible sensing mechanism

**Fig. 6:**
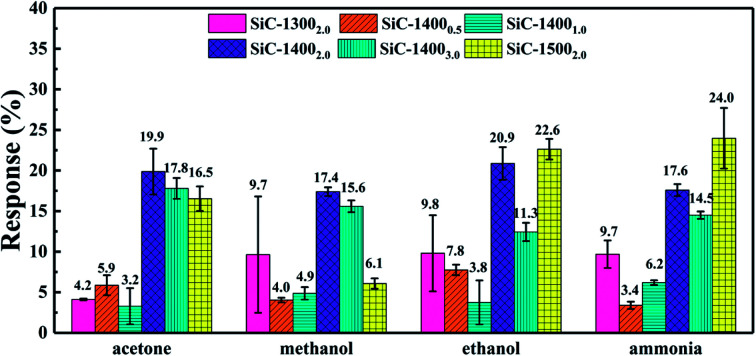
Response towards 500 ppm acetone, methanol, ethanol and ammonia under 500 °C of all SiC NSs samples.

Fig. 6 summarizes the responses towards acetone, ethanol, methanol and ammonia of SiC-1300_2.0_ SiC-1400_0.5_, SiC-1400_1.0_, SiC-1400_2.0_, SiC-1400_3.0_ and SiC-1500_2.0_ under 500 °C. Among all those samples, the SiC-1400_2.0_, SiC-1400_3.0_ and SiC-1500_2.0_ show significantly higher responses than SiC-1300_2.0_, SiC-1400_0.5_ and SiC-1400_1.0_. Particularly, SiC-1500_2.0_ possesses considerable responses towards. From the characterization results, the residual rGO composition in SiC-1300_0.5_, SiC-1400_0.5_ and SiC-1400_1.0_ is relatively high. Meanwhile, the SiC-1400_1.0_ sample, containing both SiC and rGO phase, surprisingly shows worse response than SiC-1400_0.5_. Thus, we may infer that large amount of rGO inside SiC NSs could significantly decrease their gas sensing properties. In order to obtain satisfactory SiC NSs samples, the heating time should be lengthened adequately to remove rGO composition as much as possible.

**Fig. 7:**
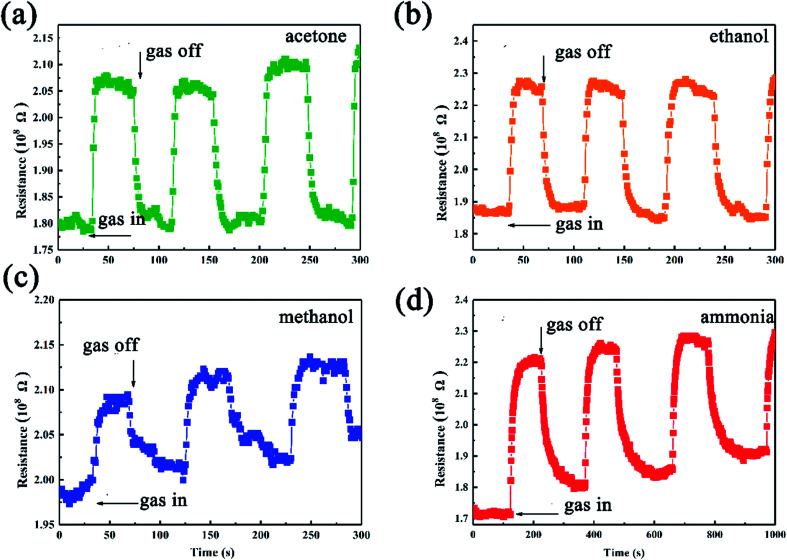
Gas sensing results of SiC-1500_2.0_-based gas sensor towards (a) acetone, (b) ethanol, (c) methanol and (d) ammonia tested under 500 °C.

The as-synthesized SiC NSs gas sensor possesses good ability to detect polar gases under high temperature, which makes it a good candidate to be applied into harsh environments. Fig. 7 shows the dynamic response behavior towards 500 ppm methanol, ethanol, acetone and ammonia of the SiC-1500_2.0_. The testing temperature was set as 500 °C. Upon the induce of all the four gases, the resistance of SiC-1500_2.0_ increases, while after exposing to air it decreases, showing a p-type sensing behavior. All the procedure was repeated for three times, and the testing results indicated that our SiC-1500_2.0_ sensor possesses a good reproducibility.

**Fig. 8:**
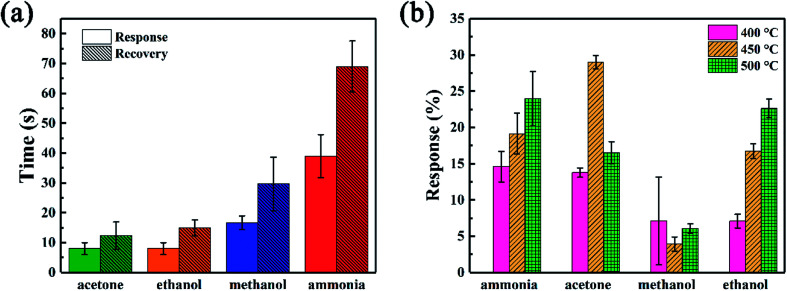
(a) Response and recovery time under 500 °C and (b) response value under different temperatures towards 500 ppm different gases of SiC-1500_2.0_ sensor.

The response and response/recovery time are both important parameters to evaluate the performance of a gas sensor. High response and fast response/recovery time could make the sensor be an excellent candidate for rapid detection of hazardous gases.^48^ To directly show these parameters of SiC-1500_2.0_-based gas sensors, the response/recovery time under 500 °C and response value under 400–500 °C towards 500 ppm different gases were illustrated in Fig. 8. Among those gases, our SiC-1500_2.0_ shows the lowest response (∼8 s) and recovery (∼12 s) time towards acetone and ethanol, while the longest response (∼40 s) and recovery (∼75 s) time towards ammonia. It is worth noticing that compared with most reported ethanol sensing materials, our SiC-1500_2.0_ gas sensor shows faster response/recovery time than most of them, making it possible to be an excellent fast detecting gas sensor applied into harsh environments. This could be contributed from the fast carrier transport rate of two-dimensional nanosheets structure. Table 3 provides the detailed data of response/recovery time towards ethanol of our SiC-1500_2.0_ and other ethanol sensors in literature. As for the value of response, this sensor also shows high response under high temperature towards ammonia, acetone and ethanol. Particularly, the SiC-1500_2.0_ sensor performs best when applied at 450 °C, where the response towards ammonia, acetone, methanol and ethanol is 19.14%, 29.03%, 3.94% and 16.76%, respectively.

**Table 3:** Comparison of the response and recovery time among different ethanol sensing materials

Sensor type	Operating temperature (°C)	Ethanol concentration (ppm)	Response time (s)	Recovery time (s)
Fe_2_O_3_ nanoporous network^49^	450	100	2	75
SnO_2_ nanowires^50^	360	100	1–10	10–100
Fe_2_O_3_@SnO_2_ nanowires^51^	300	200	∼75	∼200
Flower-like ZnO nanorods^52^	400	50	10	16
SiC NSs in this work	500	500	8	12

**Fig. 9:**
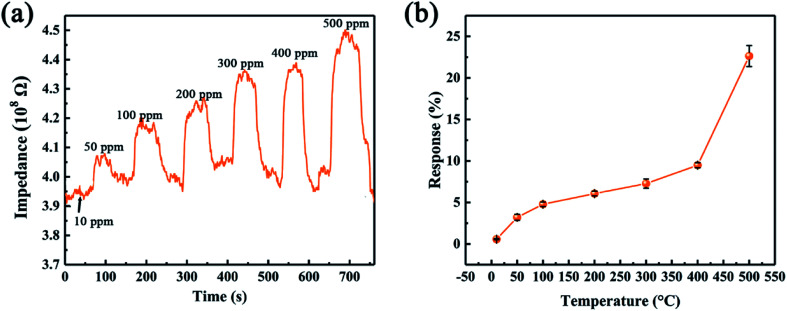
(a) Dynamic responses of SiC-1500_2.0_ toward various ethanol concentration at 500 °C. (b) SiC-1500_2.0_ sensor response toward 500 ppm ethanol under different temperature.

Since the leak of ethanol could cause severe fire accident, we further investigate the gas sensing of the device as a function of ethanol concentration. Fig. 9 compares the gas sensing performance of SiC-1500_2.0_ sensor towards 10–500 ppm ethanol. It can be observed that the device shows p-type behavior towards all ethanol concentration. As the concentration increases, the sensor response also rises up (Fig. 9b). Compared with other already reported two-dimensional materials, our limits of detection (10 ppm) of SiC-1500_2.0_ can be comparable with them,^53,54^ suggesting that this material has the ability to perform well in gas sensing fields.

**Fig. 10:**
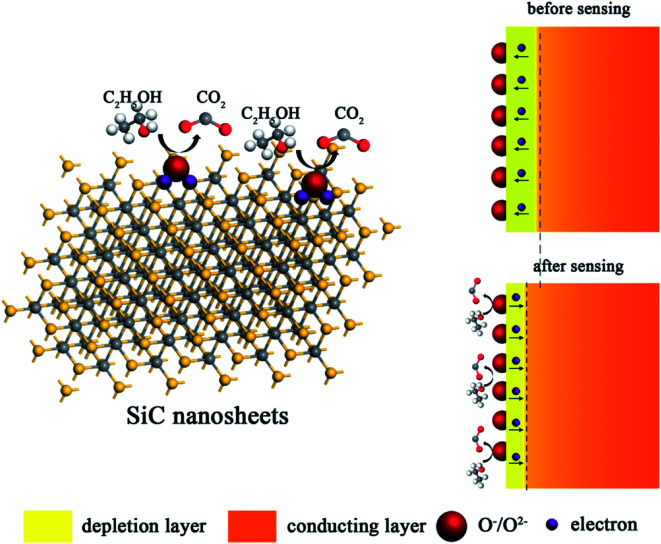
Schematic illustration of a possible sensing mechanism for SiC NSs (taking ethanol for an example). The electrons can be transferred inside the conducting layer of SiC NSs when the reducing gas molecules are adsorbed on the surface, resulting in a change of gas sensing material.

Up to date, the sensing mechanism of non-oxide-based materials is still controversial. Here, we believe that the response towards different gas molecules is originated from the functional groups and defects of SiC NSs. As shown in Fig. 10, in air, the surface of SiC NSs would form deionized oxygen (O^−^, O_2_^−^, *etc.*) due to the partially charged functional groups from oxide layer, forming a depletion layer on the surface. When the gas molecules are adsorbed, the strong force from hydrogen bonds or chemical bonds with functional groups or defects would force electrons be transferred to the SiC NSs, narrowing the thickness of depletion layer, improving the concentration of electrons and changing the resistance of gas sensing material. Taking ethanol as an example, the possible reactions are processed as follows:^55^

3 C_2_H_5_OH + O^−^/O_2_^−^ → C_2_H_4_O + H_2_O + e^−^

4 C_2_H_4_O + O^−^/O_2_^−^ → CO_2_ + H_2_O + e^−^

### 3.3 A proposal mechanism for n–p transition of SiC NSs under different temperatures

**Fig. 11:**
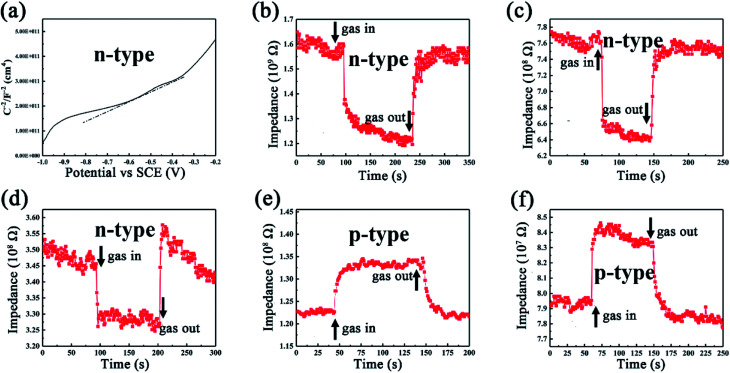
(a) Mott–Schottky plot of SiC-1500_2.0_ device under room temperature. Dynamic response towards 100 ppm ammonia under (b) 250 °C, (c) 300 °C, (d) 350 °C, (e) 400 °C and (f) 450 °C.

During the gas sensing test, an n–p transition phenomenon was found as the testing temperature increases. First, Mott–Schottky plot was conducted in order to characterize the intrinsic properties of SiC-1500_2.0_ sensor under room temperature. It is widely known that the slope of linear region of Mott–Schottky plot can be used to judge the type of a semiconductor.^56^ Accordingly, our SiC-1500_2.0_ sensor shows a positive slope in linear region of Mott–Schottky plot (Fig. 11a), which indicates that it is an n-type semiconductor under room temperature. Since our testing gases (acetone, ethanol, methanol and ammonia) are all reducing type, the gas molecules should provide electrons when adsorbed on the surface of n-type SiC NSs, resulting in the decline of resistance. However, our dynamic response curves (Fig. 7) all show p-type characteristics under 500 °C testing temperature, which suggests that the type of SiC NSs sensor may be changed when it is heated under high temperature. To carefully observe this phenomenon, the dynamic response/recovery curves towards ammonia for SiC-1500_2.0_ sensor are taken under 250–450 °C, as can be seen in Fig. 11b–f. The response curves illustrate that the SiC-1500_2.0_ sensor is still n-type below 350 °C, but interestingly changes to p-type after 400 °C. To the authors' knowledge, the n–p type conversion during gas sensing tests is already detailedly investigated towards metal oxides, such as ZnO and V_2_O_5_.^57,58^ Those authors believe that the oxygen vacancy on the surface of metal oxides play an important part in gas sensing performance, which could cause the change of electronic structure and finally reverse the type of semiconductor. Nevertheless, we believe that it is not proper to use oxygen vacancy theory to explain the n–p transition of SiC NSs, since it is hard to form such vacancy inside SiC. Thus, it is necessary to study the change under high temperature condition of our SiC NSs.

**Fig. 12:**
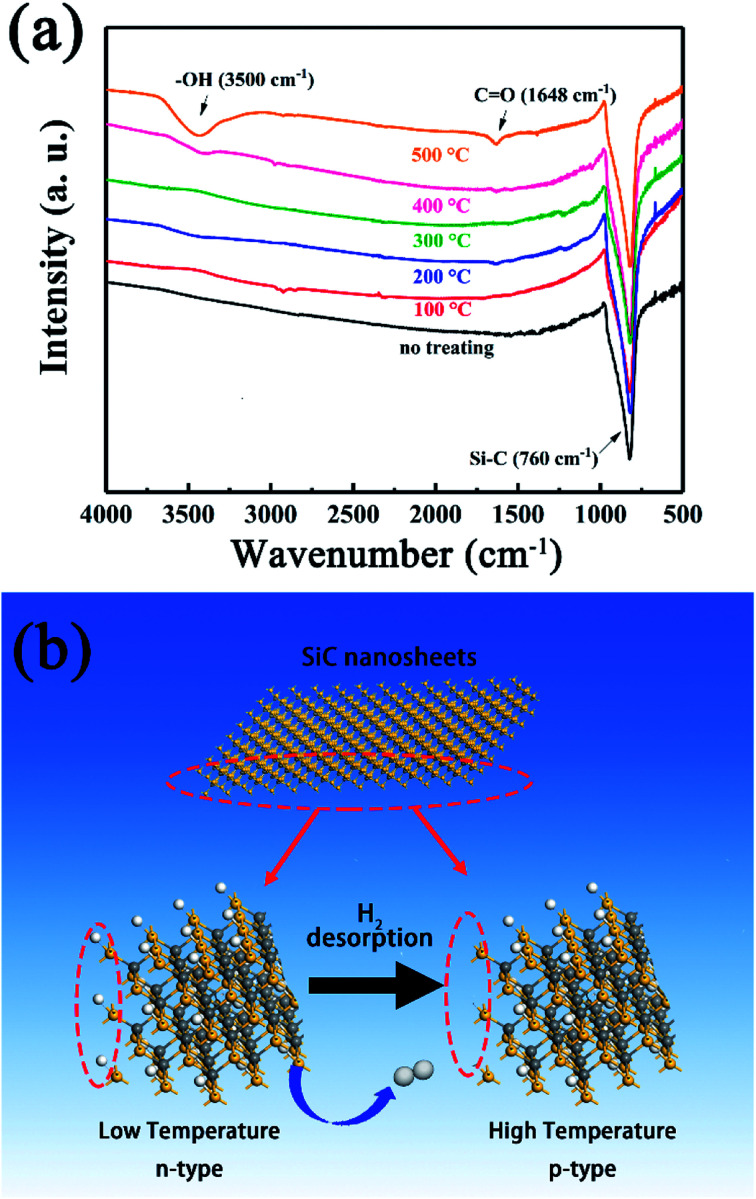
(a) FT-IR spectra obtained at room temperature of SiC-1500_2.0_ annealed at different temperature. (b) Schematic illustration of the n–p conductivity transition at high temperature.

To achieve this, the SiC-1500_2.0_ sample was heated under 100–500 °C again for 0.5 h in air to simulate the gas sensing testing condition and naturally cooled down to room temperature. Then the FT-IR spectra were used to investigate the variation of SiC-1500_2.0_. As shown in Fig. 12a, the amount of O-containing groups (C=O and –OH) increases as the annealing temperature rises up. Particularly, the sample annealed at 500 °C possess the most apparent vibration peak contributing to hydroxyl group. Since the hydroxyl group is hard to detect under such high temperature, the C=O as well as the –OH might be formed during the cooling process. King *et al.* have reported that the Si–H and Si–CH_3_ groups can be dissociated at high temperature, with a desorption of H_2_.^59–61^ During this process, the Si dangling bond can be formed, and absorb water or oxygen in air, resulting in the formation of –OH and C=O groups. From these results, a possible n–p conversion mechanism could be inferred as follows.

Accordingly, a proposed n–p conductivity transition mechanism is explained as follows (Fig. 12b). At room temperature, the residual rGO makes SiC NSs show an n-type conductivity behavior.^62^ However, as the testing temperature rises up, the Si–H and Si–CH_3_ groups on the surface of SiC NSs begin to be dissociated, leading to the desorption of H_2_ and the formation of Si dangling bonds. The decrease of H atoms could reduce the amount of electron of SiC NSs, resulting in the variation of majority carrier and the change of n–p conduction.^63^ Meanwhile, the residual rGO could be decomposed easily at high temperature, reducing the amount of n-type dopant. Therefore, an n–p conductivity conversion happens with the rising testing temperature.

## 4. Conclusions

In this paper, we have demonstrated the ability of SiC NSs to detect a series of hazardous gases in harsh environments, which proves the results of previous theoretical calculation. By controlling heating parameters, SiC NSs with different structure and composition could be obtained. The SiC NSs sensor could successfully detect acetone, methanol, ethanol and ammonia under high temperature. Meanwhile, an n–p conductivity transition is observed, and it is mainly due to the formation of oxygen-contained functional groups on the surface of SiC NSs at high temperature. This novel SiC NSs sensor opens a new direction for applying SiC materials into gas sensing fields. Furthermore, by modifying with metal or metal oxide dopant, the SiC NSs may also show excellent gas sensing performance towards other gases.

## Conflicts of interest

There are no conflicts to declare.

## Supplementary Material

RA-008-C8RA02164C-s001
