# Supplementary material for: High temperature gas sensing performances of silicon carbide nanosheets with an n–p conductivity transition
Source: RSC Adv. 2018 Apr 12;8(25):13697–707. doi: 10.1039/c8ra02164c (PMC9079785; doi:10.1039/c8ra02164c)
Supplement: RA-008-C8RA02164C-s001 [file RA-008-C8RA02164C-s001.pdf]

Electronic Supplementary Material (ESI) for RSC Advances.
This journal is © The Royal Society of Chemistry 2018

Supporting Information

High temperature gas sensing performances of silicon carbide nanosheets with an n-p conductivity transition

Lian Sun[a], Cheng Han[a], Nan Wu[a], Bing Wang [a] *, Yingde Wang[a] *

[a]*Science and Technology on Advanced Ceramic Fibers and Composites Laboratory, National University of Defense Technology, Changsha 410073, China*

***Corresponding Author**: Tel.: +86 731 84574196. E-mail: cfcwb01@163.com (B. Wang) wangyingde@nudt.edu.cn (Y. D. Wang)

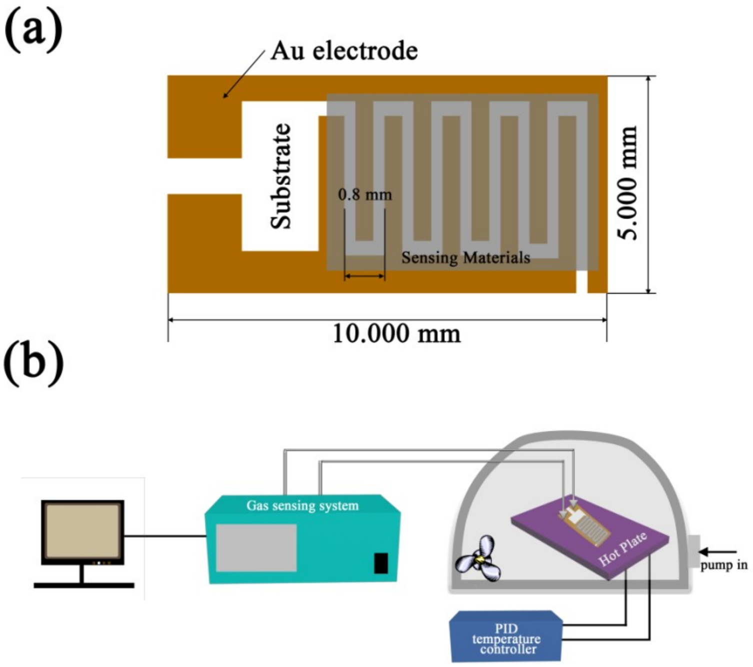

Figure S1 Schematic illustration of (a) gas sensing device after spin-coated and (b) testing system.

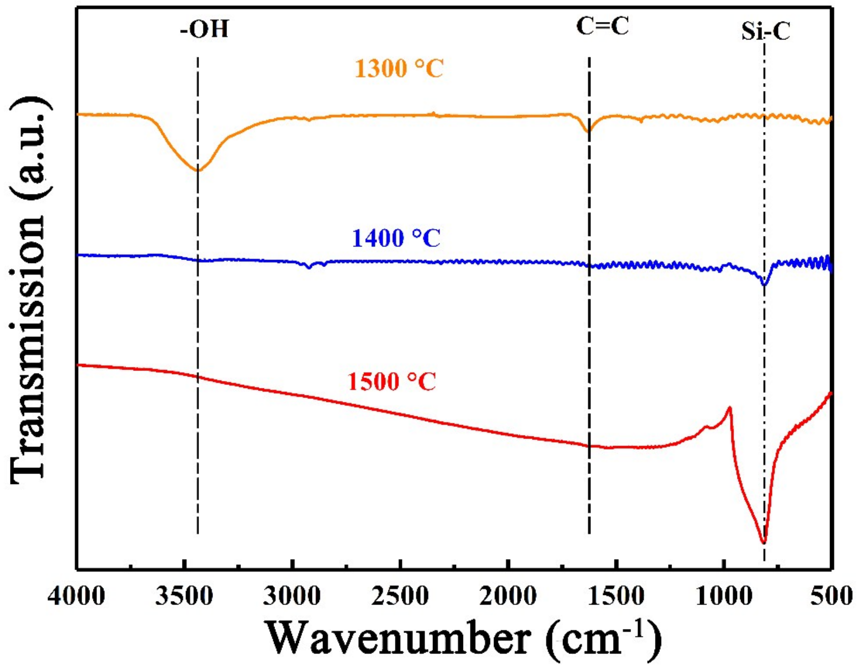

Figure S2 FT-IR spectra of SiC NSs heated at different temperatures for 2 h.

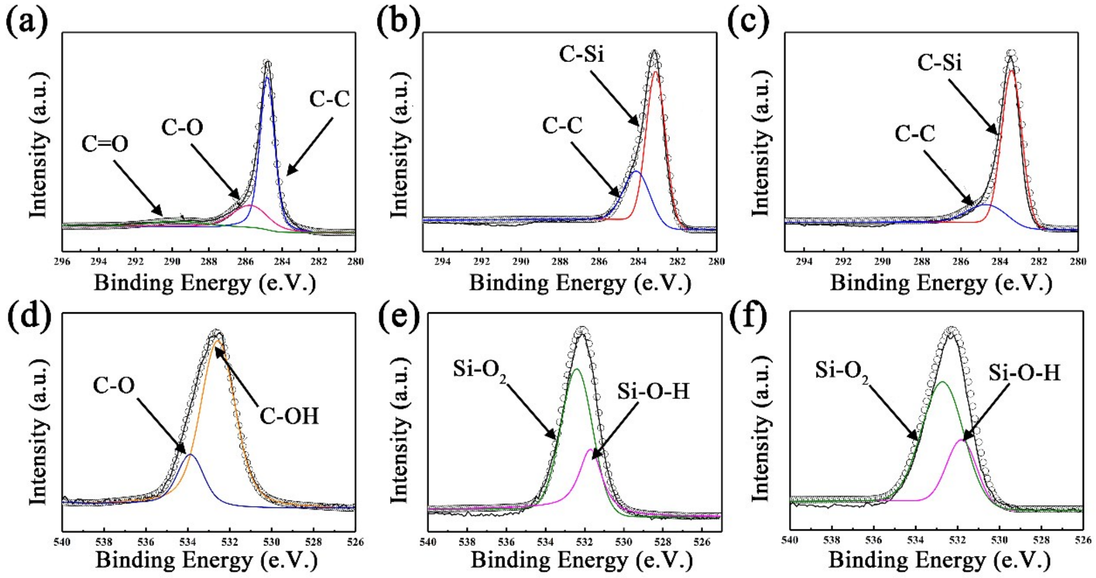

Figure S3 High resolution (a)-(c) C_{1s} and (d)-(f) O_{1s} XPS peaks of SiC NSs heated for 2 h at (a) (d) 1300 °C, (b) (e) 1400 °C and (c) (f) 1500 °C.

The high-resolution C_{1s} peaks of SiC-$1400_{2.0}$ and SiC-$1500_{2.0}$ give peaks at 283.1 and 284.6 eV, which is ascribed to C-Si and C-C bond, respectively [1].. On the contrast, the C_{1s} high resolution spectrum of SiC-$1300_{2.0}$ mainly consists of C-C (284.6 eV), C-O (285.8 eV) and C=O (289.6 eV) bond, implying that the main composition of this sample is carbon phase [2-3]. Correspondingly, the deconvolution of O_{1s} peak gives Si-O_2 and Si-O-H bond (532.7 and 531.8 eV, respectively) above 1400 °C, while only C-OH (532.4 eV) and C-O (533.9 eV) bond can be found in the O_{1s} peak of SiC-1300_2 [4].

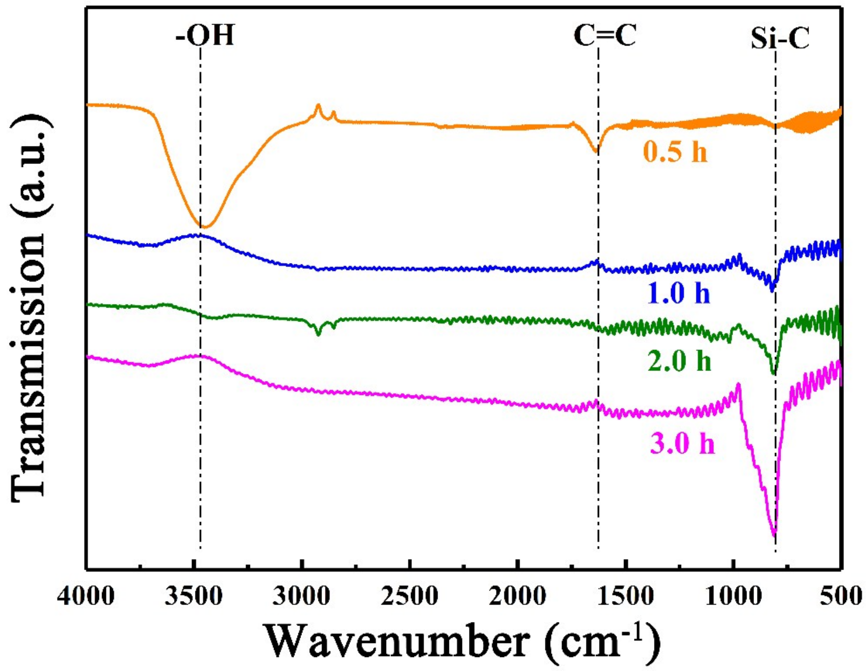

Figure S4 FT-IR spectra of SiC NSs heated at 1400 °C for different time.

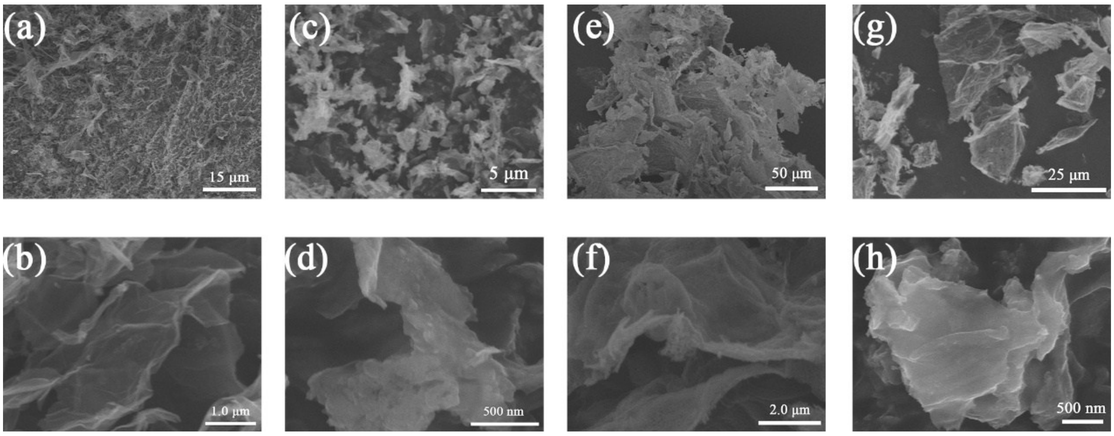

Figure S5 SEM images of SiC NSs synthesis under 1400 °C for (a) (b) 0.5 h, (c) (d) 1.0 h, (e) (f) 2.0 h, (g) (h) 3.0 h.

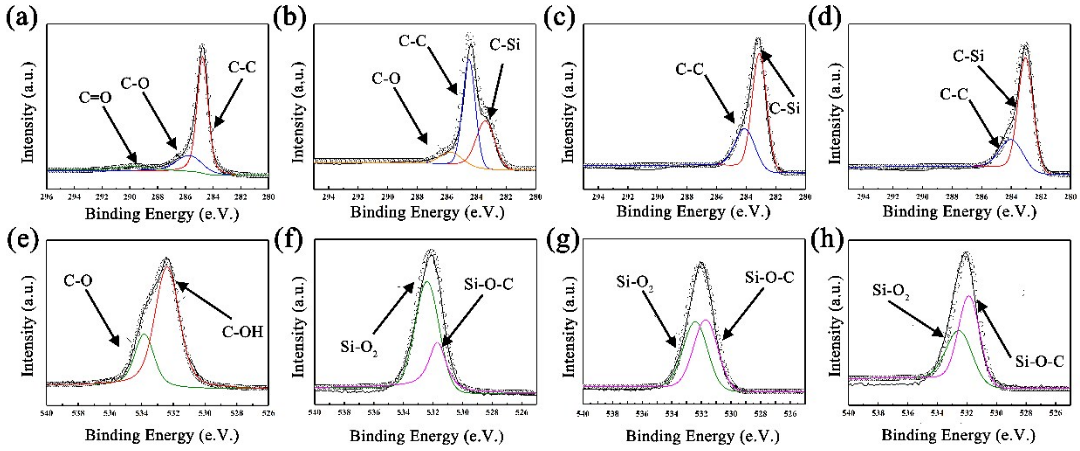

Figure S6 High resolution (a)-(d) C_{1s} and (e)-(h) O_{1s} XPS peaks of SiC NSs heated at 1400 °C for (a) (e) 0.5 h, (b) (f) 1.0 h, (c) (g) 2.0 h, (d) (h) 3.0 h.

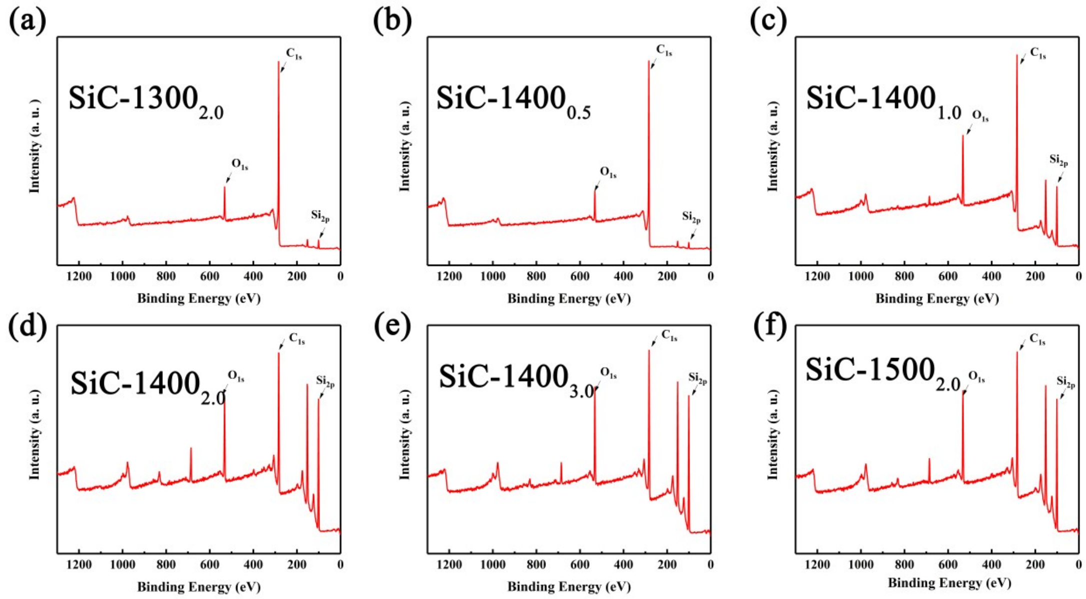

Figure S7 XPS survey data of different SiC NSs samples.

Table S1 Positions and FWHM of the deconvoluted Si_{2p} peaks of samples containing SiC

Sample	SiC-$1400_{1.0}$	SiC-$1400_{2.0}$	SiC-$1400_{3.0}$	SiC-$1500_{2.0}$
Position of Si-C bond	100.8 eV	100.9 eV	100.9 eV	101.0 eV
FWHM of Si-C bond	1.22	1.09	1	0.89
Position of Si-O bond	101.7 eV	101.7 eV	101.7 eV	101.7 eV
FWHM of Si-O bond	1.2	1.1	1.22	1.22

References:

[1] F. Ténégal, A. Gheorghiu de la Rocque, G. Dufour, C. Sénémaud, B. Doucey, D. Bahloul-Hourlier, P. Goursat, M. Mayne and M. Cauchetier, *J. Electron Spectrosc. Relat. Phenom.,* 2000, **109**, 241-248.

[2] S. Golczak, A. Kanciurzewska, M. Fahlman, K. Langer and J. J. Langer, *Solid State Ionics,* 2008, **179**, 2234-2239.

[3] A. P. Dementjev, A. de Graaf, M. C. M can de Sanden, K. I. Maslakov, A. V. Naumkin and A. A. Serov, *Diamond Relat. Mater.,* 2000, **9**, 1904-1907.

[4] B. Wang, Y. D. Wang, Y. P. Lei, S. Xie, N. Wu, Y. Z. Gou, C. Han, Q. Shi and D. Fang, *J. Mater. Chem. C,* 2016, **4**, 295-304.
